# Supplementary material for: Enhancing polyol/sugar cascade oxidation to formic acid with defect rich MnO2 catalysts
Source: Nat Commun. 2023 Jul 26;14:4509. doi: 10.1038/s41467-023-40306-w (PMC10372030; doi:10.1038/s41467-023-40306-w)
Supplement: Supplementary file 1 — Supplementary Information [file 41467_2023_40306_MOESM1_ESM.pdf]

Supplementary Information

Enhancing Polyol/Sugar Cascade Oxidation to Formic Acid with Defect Rich MnO_2 Catalysts

Hao Yan,[1,2#] Bowen Liu,[3,#] Xin Zhou,[1,4#] Fanyu Meng,[1] Mingyue Zhao,[1] Yue Pan,[1] Jie Li,[1] Yining Wu,[5] Hui Zhao,[1] Yibin Liu,[1,*] Xiaobo Chen,[1] Lina Li,[6] Xiang Feng,[1,*] De Chen,[7] Honghong Shan,[1] Chaohe Yang[1] and Ning Yan[2*]

[1]State Key Laboratory of Heavy Oil Processing, China University of Petroleum (East China), Qingdao 266580, China

[2]Department of Chemical and Biomolecular Engineering, National University of Singapore, Engineering Drive 4, 117585, Singapore

[3]Department of Chemistry, University of Liverpool, Crown Street, L69 7ZD Liverpool, United Kingdom

[4]College of Chemistry and Chemical Engineering, Ocean University of China, Qingdao, Shandong 266100, China

[5]School of Petroleum Engineering, China University of Petroleum (East China), Qingdao 266580, China

[6]Shanghai Synchrotron Radiation Facility, Shanghai Advanced Research Institute, Chinese Academy of Sciences, Shanghai 201204, China

[7]Department of Chemical Engineering, Norwegian University of Science and Technology, Trondheim 7491, Norway

# Hao Yan, Bowen Liu and Xin Zhou contributed equally to this work.

Corresponding author

Correspondence to: Xiang Feng (xiangfeng@upc.edu.cn); Yibin Liu (liuyibin@upc.edu.cn); Ning Yan (ning.yan@nus.edu.sg)

Content

1. Experimental section 4
2. Supplementary Tables 7
Supplementary Table 1. Unit cell parameter, particle size and micro strain from PXRD analysis of MnO_2-P. 7
Supplementary Table 2. Unit cell parameter, particle size and micro strain from PXRD analysis of MnO_2-D. 7
Supplementary Table 3-1. EXAFS fitting parameters at the Mn K-edge for MnO_2-P, MnO_2-T and MnO_2-D (S_0^2=0.777 for Mn) 8
Supplementary Table 3-2. EXAFS fitting parameters at the Mn K-edge for (β-)MnO_2-H and (β-)MnO_2-H (S_0^2=0.777 for Mn) 8
Supplementary Table 4-1. Peak-fitting results of Mn 2*p* of XPS spectra for the MnO_2-P and MnO_2-D catalysts. 9
Supplementary Table 4-2. Peak-fitting results of O 1*s* of XPS spectra for the MnO_2-P and MnO_2-D catalysts. 9
Supplementary Table 5-1. Quantitative analysis of NH_3-TPD 10
Supplementary Table 5-2. Quantitative analysis of CO_2-TPD 10
Supplementary Table 5-3. Quantitative analysis of O_2-TPD 10
Supplementary Table 6-1. Oxidation of glycerol and other substrates over Mn-based catalysts 11
Supplementary Table 6-2. Oxidation of glycerol over MnO_2 catalysts 13
Supplementary Table 7. Oxidation of glycerol over MnO_2-D catalysts and reported catalysts..... 14
Kinetics determination..... 16
3. Supplementary Figures..... 17
Supplementary Fig. 1 Supplemental HRTEM images for the (a) MnO_2-P and (b) MnO_2-D catalysts...... 17
Supplementary Fig. 2-1 (a) Visualisation of the associated crystal structure. (b) Wavelet transform map of different coordination atoms. Fourier transform of Mn K-edge extended EXAFS oscillations and the first-order derivatives of Mn K-edge XANES of (c) MnO_2-T and (d) β-MnO_2. 18
Supplementary Fig. 2-2. (a) XRD patterns, (b) NH_3-TPD and (c) CO_2-TPD of α-MnO_2, β-MnO_2 and γ-MnO_2 with high or low coordination number. 20
Supplementary Fig. 3 Mn electronic structure analysis from XANES spectra. 21
Supplementary Fig. 4 EPR spectra of the MnO_2-P and MnO_2-D catalysts. 22
Supplementary Fig. 5 (a)XRD, (b) UV-vis, (c) Mn 2*p* and (d) O 1*s* XPS spectra of the fresh MnO_2-D and used MnO_2-D catalysts. (e) NH_3-TPD and CO_2-TPD of spent MnO_2-P and MnO_2-D. (f) Pyridine (py-)IR of MnO_2-P and MnO_2-D with and without water. (g) IR spectra of the spent MnO_2-P, spent MnO_2-D, fresh MnO_2-P and spent MnO_2-D..... 23
Supplementary Fig. 6 (a) Nitrogen adsorption-desorption isotherms and (b) pore size distributions of the MnO_2-P and MnO_2-D catalysts. 25
Supplementary Fig. 7 Catalytic performance of glycerol oxidation on (a) MnO_2-P and (b) MnO_2-D catalysts as a function of reaction time (experiment conditions: 25 mL aqueous phase solution (0.1 M), 0.5 g NaOH, 0.1 g

catalyst, 1 MPa O_2, 120^oC; Glyceric acid, propanedioic acid, glycolic acid, oxalic acid and glycerol are abbreviated as GLYA, TA, GLYOA, OA and FA respectively). (c) Catalytic stability of the MnO_2-P and MnO_2-D under multiple cycle test conditions. 26

Supplementary Fig. 8 A schematic diagram of ethanol pulse adsorption of the MnO_2-P and MnO_2-D catalysts. 27

Supplementary Fig. 9 Oxidation mechanism study based on the isotope labelling experiments over (a) MnO_2-D and (b) $Mn^{18}O_2$-D. [Reaction conditions: 25 mL aqueous phase solution (0.1 M), 0.1 g catalyst, 1 MPa O_2, 140^oC, 4 h].......... 28

Supplementary Fig. 10 Apparent activation energy calculated from the power function type reaction kinetic equation over the MnO_2-P and MnO_2-D.......... 29

Supplementary Fig. 11 DFT calculation models of (a) the MnO_2-P (Penta) and MnO_2-D (Tri). 30

Supplementary Fig. 12-1 Free energy diagrams for the oxidation of glycerol to formic acid on the MnO_2-P (Penta) [blue] and MnO_2-D (Tri) [red] 31

Supplementary Fig. 12-2 Configuration diagrams of reactants, transition states, and products on the MnO_2-P (Penta) [blue] and MnO_2-D (Tri) [red] (Step-1 to Step-4).......... 32

Supplementary Fig. 12-3 Configuration diagrams of reactants, transition states, and products on the MnO_2-P (Penta) [blue] and MnO_2-D (Tri) [red] (Step-5 to Step-8).......... 33

Supplementary Fig. 12-4 Configuration diagrams of reactants, transition states, and products on the MnO_2-P (Penta) [blue] and MnO_2-D (Tri) [red] (Step-9 to Step-10). Mulliken charge (|e|) distribution of the rate-determining step (Step-10) in the MnO_2-P (Penta) and MnO_2-D (Tri).......... 34

1. Experimental section

Reagents

Hydrochloric acid (HCl, 37 wt%, Sinopharm Chemical Reagent Co., Ltd)

Potassium Permanganate ($KMnO_4$, AR, Sinopharm Chemical Reagent Co., Ltd)

Manganese(II) Chloride Tetrahydrate ($MnCl_4$•4H_2O, AR, Sinopharm Chemical Reagent Co., Ltd)

Manganese(II) Acetate Tetrahydrate ($(CH_3COO)_2Mn \cdot 4H_2O$, AR, Sinopharm Chemical Reagent Co., Ltd)

Ammonium Sulfate ($(NH_4)_2SO_8$, AR, Sinopharm Chemical Reagent Co., Ltd)

γ-manganese dioxide (γ-MnO_2, McLean reagent)

Ammonium Oxalate Monohydrate ($(NH_4)_2C_2O_4 \cdot H_2O$, AR, Sinopharm Chemical Reagent Co., Ltd)

Glycerol ($C_3H_8O_3$, 99.5%+, Sinopharm Chemical Reagent Co., Ltd)

Ethylene glycol ($C_2H_6O_2$, 99.5%+, Sinopharm Chemical Reagent Co., Ltd)

Meso-Erythritol ($C_4H_{10}O_4$, 99%, Sinopharm Chemical Reagent Co., Ltd)

D-Sorbitol ($C_6H_{14}O_6$, 98+%, Adamas)

Xylitol ($C_5H_{12}O_5$, 99+%, Sinopharm Chemical Reagent Co., Ltd)

Synthesis methods of other MnO_2 materials

Tetra-coordinated α-MnO_2 (MnO_2-T)

The $KMnO_4$ (0.79 g) and $(CH_3COO)_2Mn \cdot 4H_2O$ (1.8 g) were dissolved in 50 mL of deionized water and stirred for 30 min. The solution was transferred in an autoclave, sealed, and placed in an oven at 140 °C for 2 h. After cooling to room temperature, the residue was washed with water thrice and dried at 60 °C in an oven. Finally, the sample was calcined to obtain the MnO_2-T catalyst in muffle furnace at 400 ^{o}C for 1 h.

β-MnO_2 [(β-)MnO_2-H and (β-)MnO_2-L]

The $(NH_4)_2SO_8$ (19.494 g) and $MnCl_4$•4H_2O (17.019 g) were mixed in 400 mL water and treated in the Teflon-sealed autoclave at 160°C for 12 h. The as-formed sample was calcinated at 400°C for 4h to obtain

(β-)MnO_2-H. For the (β-)MnO_2-L, the $MnCl_4$•$4H_2O$ and $KMnO_4$ (total amount of 20 mmol/L) was stirred for 2 h and then transferred into the Teflon-sealed autoclave at 160°C for 12 h..

γ-MnO_2 [(γ-)MnO_2-H and (γ-)MnO_2-L]

The $(NH_4)_2SO_8$ (9.006 g) and $MnCl_4$•$4H_2O$ (7.916 g) were mixed in 400 mL water and treated in the Teflon-sealed autoclave at 90°C for 24 h [(γ-)MnO_2-H]. For the (γ-)MnO_2-L, it was purchased from McLean reagent.

XPS study

The optimal spatial resolution, optimal energy resolution and (large area) energy resolution of monochromator are 20 microns, 0.45 eV and 400000 cps (FWHM ≤ 0.5 eV), respectively. The excitation source was Al Kα ray (hv=1486.6 eV), the working voltage was 12.5 kV, the filament current was 16 mA, the spot beam was 400 μm, and the signal accumulation was carried out for 10 cycles. The full spectrum of the test pass-energy is 100 eV, the narrow spectrum is 20 eV, the step size is 0.1 eV, and the residence time is 40-50 ms. Test steps: the powder is pressed and prepared into the sample plate, then put into the instrument for vacuum testing. Several basic rules of XPS peak-splitting fitting are as follows: 1) The binding energy of different chemical state of each element reference online database at http://srdata.nist.gov/xps/selEnergyType.aspx. 2) The splitting intensity ratio of p, d and f levels is certain, $p_{3/2}$:$p_{1/2}$=2:1; $d_{5/2}$:$d_{3/2}$=3:2, $f_{7/2}$:$f_{5/2}$=4:3. This rule should be followed during peak fitting. 3) For the energy level with splitting (p, d, f), the distance between the two splitting orbitals is basically fixed. For example, the difference between Mn $p_{3/2}$ and Mn $p_{1/2}$ in the same state is about 11.25 eV. The distance between the two orbitals of energy level splitting will be different in specific chemical states. 4) For a certain metal, the full width at half maxima (FWHM) of the valence peak of the same orbital in different samples should be similar; for the split orbit of the same element, FWHM should be as close to the same as possible. Generally, FWHM is no more than 2.7 eV. 5) For the same instrument and instrument parameters, Gaussian-Lorentzian (GL) ratio of each data shall be consistent. Generally, GL ration is about 20%.

In situ FT-IR study

For the in situ FTIR of glycerol oxidation, the catalysts were impregnated with glycerol via an ex-situ wet impregnation method. Certain amount of catalysts, typically 300 mg, was added to a 5 mL mixed solution of glycerol and sodium hydroxide (0.5 mol/L) in order to pre-absorb quantitative glycerol and NaOH. The resulting slurry was mixed in a shaker with continuous magnetic stirring for 4 h at room temperature. The pre-absorbed sample was collected after low speed centrifugalization then dried through vacuum freeze-drying for 24 h. After that, spectra were collected over the treated catalysts as backgrounds at 25 °C for 1h (record background) in N_2 (30 mL/min). Subsequently, the spectra of glycerol pre-absorbed samples

were recorded at room temperature to characterize the adsorption form, then 5 vol% O_2 and N_2 mixture gas (30 mL/min) was introduced in the IR cell with the increase of temperature (200°C). The final spectrum was obtained by subtracting the spectrum of the sample from the adsorbed sample spectrum. The water resistance behavior of the catalyst's Lewis Acid Sites was examined utilizing a NEXUS Fourier transform infrared (FTIR) spectrometer from Thermo Electron. Self-supporting disks (20 mm diameter, approximately 20 mg) of the samples were compressed and placed in an infrared (IR) cell that was affixed to a closed circulation system. To eliminate physically adsorbed water, the disks were dehydrated at 473 K under vacuum for one hour. Afterwards, the dehydrated disks were introduced to saturated water vapor (roughly 20 Torr) at room temperature for one hour, followed by a 5-minute evacuation to eliminate weakly adsorbed water and essentially generate hydrated samples. Subsequently, the valve of the pyridine cell was opened, and the pyridine vapor was introduced and allowed to adsorb for one hour. After the adsorption had finished, the spectra of the samples were obtained at varying temperatures, with the signal at 1450 cm^{-1} representing the L acid site and the signal near 1650 cm-1 representing water adsorbed by the sample.

2. Supplementary Tables

Supplementary Table 1. Unit cell parameter, particle size and micro strain from PXRD analysis of MnO_2-P.

MnO_2 ($Mn^{3.93+}O_{1.94}^{2-}$)			Space group		*I*4/*m* (87)	
Lattice parameter (Å/ Å^3)			a:9.8037(2)	c:2.8550(5)	V:274.383(21)	
Coordinate	x	y	z	occupancy	isotropic temperature factor	M-O distance(Å)
O1	0.1798(4)	0.2032(5)	0	0.95(2)	3.9711(212)	1.64(3)
O2	0.1707(5)	0.4606(4)	0	1.00(1)	3.6714(132)	1.89(3)
Mn1	0.3394(2)	0.1576(2)	0	0.99(1)	1.8548(168)	-
R_wp	11.8515					

Note: Both the α-MnO_2 and OMS-2 zeolite have the same space group (I4/m), ICP-OES has been used to measure the K+ content in the synthesized samples. The ICP-OES results indicated that, the K+ in both two synthesized samples (MnO_2-P: 1.8 wt% and MnO_2-D: 1.9 wt%) are lower than 2%, thus, the K+ at 4e was not considered during the Rietveld refinements. All the Rietveld refinements gave satisfactory agreement factors.

Supplementary Table 2. Unit cell parameter, particle size and micro strain from PXRD analysis of MnO_2-D.

MnO_2 ($Mn^{3.64+}O_{1.80}^{2-}$)			Space group		*I*4/*m* (87)	
Lattice parameter (Å/Å^3)			a:9.8179(12)	C:2.8467(30)	V:274.395(71)	
Coordinate	x	y	z	occupancy	isotropic temperature factor	M-O distance(Å)
O1	0.1806(9)	0.2036(9)	0	0.87(1)	6.63(10)	1.69(4)
O2	0.1633(11)	0.4584(10)	0	0.93(2)	6.01(15)	1.92(4)
Mn1	0.3456(4)	0.1606(4)	0	0.99(2)	3.45(12)	-
R_wp	9.3990					

Supplementary Table 3-1. EXAFS fitting parameters at the Mn K-edge for MnO_2-P, MnO_2-T and MnO_2-D (S_0^2=0.777 for Mn)

Sample	Shell	CN^a	R(Å)^b	σ^2(Å^2)^c	ΔE_0(eV)^d	R factor
Mn foil	Mn-Mn	12*	2.66±0.01	0.007±0.002	6.69±2.67	0.016
	Mn-O	6	1.88±0.01	0.001±0.001	-3.41±1.68	
MnO_2	Mn-Mn	4	2.87±0.01	0.002±0.0009	-8.07±1.31	0.02
	Mn-Mn	4	3.42±0.01	0.002±0.0009	-8.07±1.31	
	Mn-O	5.23±0.33	1.85±0.01	0.0013±0.001	0.77±1.45	
MnO_2-P	Mn-Mn	2.91±0.41	2.84±0.01	0.002±0.001	-4.28±1.88	0.015
	Mn-Mn	2.93±0.22	3.38±0.01	0.002±0.001	-4.28±1.88	
	Mn-O	4.20±0.51	1.89±0.01	0.00173±0.001	1.87±0.31	
MnO_2-T	Mn-Mn	2.86±0.34	2.99±0.01	0.00319±0.001	-6.61±0.78	0.006
	Mn-Mn	2.36±0.33	3.40±0.01	0.00121±0.001	-3.98±1.40	
	Mn-O	3.04±0.39	1.89±0.01	0.0012±0.0015	-0.48±1.84	
MnO_2-D	Mn-Mn	1.12±0.28	2.87±0.01	0.0005±0.0018	-0.06±0.01	0.012
	Mn-Mn	1.36±0.33	3.41±0.01	0.0005±0.0018	-0.06±0.01	

^{a}*CN*, coordination number; ^{b}*R*, distance between absorber and backscatter atoms; $^c\sigma^2$, Debye-Waller factor to account for both thermal and structural disorders; $^d\Delta E_0$, inner potential correction; *R* factor indicates the goodness of the fit. $S0^2$ was fixed to 0.777 for Mn, according to the experimental EXAFS fit of Mn foil by fixing CN as the known crystallographic value.

Supplementary Table 3-2. EXAFS fitting parameters at the Mn K-edge for (β-)MnO_2-H and (β-)MnO_2-H (S_0^2=0.777 for Mn)

Sample	Shell	CN^a	R(Å)^b	σ^2(Å^2)^c	ΔE_0(eV)^d	R factor
Mn foil	Mn-Mn	12*	2.66±0.01	0.007±0.002	6.69±2.67	0.016
	Mn-O	6	1.88±0.01	0.001±0.001	-3.41±1.68	
MnO_2	Mn-Mn	4	2.87±0.01	0.002±0.0009	-8.07±1.31	0.02
	Mn-Mn	4	3.42±0.01	0.002±0.0009	-8.07±1.31	
	Mn-O	5.35±0.80	1.88±0.01	0.00115±0.001	2.17±0.36	
(β-)MnO_2-H	Mn-Mn	3.04±0.41	2.82±0.01	0.00316±0.002	-5.88±0.26	0.006
	Mn-Mn	1.65±0.32	3.43±0.01	0.00168±0.001	-2.16±1.94	
	Mn-O	3.39±0.46	1.88±0.01	0.00129±0.001	1.97±0.21	
(β-)MnO_2-L	Mn-Mn	2.14±0.89	2.84±0.01	0.00464±0.001	-5.02±0.12	0.006
	Mn-Mn	3.35±0.56	3.43±0.01	0.00198±0.0001	-2.36±0.96	

^{a}*CN*, coordination number; ^{b}*R*, distance between absorber and backscatter atoms; $^c\sigma^2$, Debye-Waller factor to account for both thermal and structural disorders; $^d\Delta E_0$, inner potential correction; *R* factor indicates the goodness of the fit. $S0^2$ was fixed to 0.777 for Mn, according to the experimental EXAFS fit of Mn foil by fixing CN as the known crystallographic value.

Supplementary Table 4-1. Peak-fitting results of Mn 2*p* of XPS spectra for the MnO_2-P and MnO_2-D catalysts.

	Binding energy and peak position of Mn 2p			
	Mn^{2+}(eV)	Mn^{3+}(eV)	Mn^{4+}(eV)	Satellite (eV)
MnO_2-P	641.0 (13.8%)	642.2 (33.7%)	643.1 (38.3%)	644.6 (14.2%)
MnO_2-D	641.0 (23.6%)	642.0 (35.2%)	643.0 (34.1%)	644.5 (7.1%)
Used- MnO_2-D	641.3(24.1%)	642.3 (34.8%)	643.3 (33.8%)	644.8 (7.3%)

Supplementary Table 4-2. Peak-fitting results of O 1*s* of XPS spectra for the MnO_2-P and MnO_2-D catalysts.

	Binding energy and peak position of O 1s		
	O_{III} (eV)	O_{II} (eV)	O_I (eV)
MnO_2-P	532.8 (11.2%)	531.4(17.7%)	529.9 (71.1%)
MnO_2-D	533.3 (10.9%)	531.6 (27.6%)	529.4 (61.5%)
Used-MnO_2-D	533.6 (10.6%)	531.2 (24.9%)	529.9(64.5%)

Supplementary Table 5-1. Quantitative analysis of NH_3-TPD

Sample	NH_3-TPD						
	Total consumption (mmol/g)	Weak (~150℃)		Medium (~300℃)		Strong (~580℃)	
		Consumption (mmol/g)	Percentage (%)	Consumption (mmol/g)	Percentage (%)	Consumption (mmol/g)	Percentage (%)
MnO_2-P	0.36	0.24	67.3%	0.05	12.9%	0.07	19.8%
MnO_2-D	0.63	0.06	10.6%	0.28	43.8%	0.29	45.7%

Supplementary Table 5-2. Quantitative analysis of CO_2-TPD

Sample	CO_2-TPD				
	Total consumption (mmol/g)	Weak (~150℃)		Strong (~500℃)	
		Consumption (mmol/g)	Percentage (%)	Consumption (mmol/g)	Percentage (%)
MnO_2-P	2.31	1.09	47.4%	1.22	52.6%
MnO_2-D	3.96	0.25	6.5%	3.71	93.5%

Supplementary Table 5-3. Quantitative analysis of O_2-TPD

Sample	O_2-TPD		
	Weak (~200℃) (%)	Medium (~350℃) (%)	Strong (~650℃) (%)
MnO_2-P	47.0%	42.1%	10.9%
MnO_2-D	12.9%	77.6%	9.5%

Supplementary Table 6-1. Oxidation of glycerol and other substrates over Mn-based catalysts

Catalyst	Selectivity (%)					Conversion (%)	TOF (h^{-1})	Initial reaction rate ($mmol/h/g_{cat}$)	C%
	GLYA	GLYOA	OA	FA	Others				
Blank (no catalyst)	26.7	28.5	14.5	0.0	2.1	1.0	-	-	99.7
MnO_2-P[a]	7.1	14.5	21.8	23.8	31.4	63.8	9.6	1.6	99.1
MnO_2-D[b]	4.3	4.1	3.6	83.3	3.6	63.9	113.5	31.3	99.3
MnO[c]	44.4	3.6	0.0	0.0	48.9	5.9	-	-	99.8
Mn_2O_3[c]	39.7	1.6	0.0	1.1	52.8	5.1	-	9	99.8
MnO_2[c]	70.2	3.5	0.0	0.2	25.0	5.1	-	-	99.9
Ethylene glycol (EG)[d]	0.1	2.5	0.0	84.5	11.1	99.1	-	-	98.2
1,2-propanediol (PG-2)[e]	0.0	0.0	0.0	66.5	31.5	77.4	-	-	98.5
1,3-propanediol (PG-3)[e]	0.0	0.0	0.0	65.7	30.5	82.5	-	-	96.9
Erythritol (ET)[f]	0.3	0.0	3.5	95.0	0.4	99.8	-	-	99.2
Xylitol (XT)[f]	0.0	0.0	3.9	87.6	5.8	99.7	-	-	97.3
Sorbitol (ST)[f]	0.0	1.6	8.0	82.5	4.4	99.8	-	-	96.5
Formic acid (FA)[e]	0.0	0.0	0.0	-	81.5	5.5	-	-	99.0
Glycolic acid (GLYOA)[e]	0.0	-	1.5	91.5	4.0	99.1	-	-	97.0
Oxalic acid (OA)[g]	0.0	0.0	-	85.1	10.2	94.3	-	-	95.6
Glycerol (GLYA)[e]	-	10.2	2.2	80.5	2.8	99.6	-	-	95.7
Tartronic acid (TA)[g]	0.0	10.5	6.1	78.2	3.6	99.1	-	-	98.4

Note:

1) C%: carbon balance, others: hydroxypyruvate, tartronic acid, lactic acid and CO_2.

2) TOF was calculated as mole of substrate molecules converted per mole of surface atoms (Mn) per hour. When the conversions were less than 20%, the average reaction rate is equal to the instantaneous reaction rate. The metal dispersions of the MnO_2-P and MnO_2-D catalyst are 1.5% and 2.4% (measured by ethanol pulse), respectively.

3) Reaction conditions: 0.1 g catalyst, 25 mL aqueous solution of glycerol (0.1 M), 120 °C, 0.5g NaOH, 1 MPa O_2. a. MnO-P for 10 h; b. MnO_2-D for 2 h; c. reaction time for 6 h. MnO_2-D catalyst for various substrates. d. EG: 140°C, 6 h;

e. PG, FA, GLYOA and GLYA: 120°C, 6 h; f. ET, XT and ST: 100°C, 6 h; g. OA and TA: 140°C, 6 h;

5) Taking the MnO_2-D catalyst as an example, the calculation process of initial reaction rate for glycerol oxidation is as follows:

$$\text{Initial reaction rate} = \frac{N_{\text{glycerol. converted}}}{m_{\text{Catalyst}} \cdot \text{Time}} = \frac{0.1\times0.025\times10.4\%}{0.1\times0.083} = 31.3\ \text{mmol/h/g}_{\text{cat}}$$

[The detailed parameters : 0.1 M glycerol solution (25 mL), 10.4% glycerol conversion, 0.083 h reaction time, 0.1 g catalyst]

6) Taking the MnO_2-D catalyst as an example, the calculation process of TOF value for glycerol oxidation is as follows:

$$\text{TOF} = \frac{N_{\text{glycerol. converted}}}{N_{\text{Surface active site}} \cdot \text{Time}} = \frac{0.025\times0.1\times10.4\%}{0.1\times11.5\times10^{-3}\times0.024\times0.083} = 113.5\ h^{-1}$$

[The detailed parameters : 0.1 M glycerol solution (25 mL), 10.4% glycerol conversion, 0.1 g catalyst, 11.5 mmol/g total metal atoms, 2.4% metal dispersion, 0.083 h reaction time]

Supplementary Table 6-2. Oxidation of glycerol over MnO_2 catalysts

Catalyst	Selectivity (%)					Conversion (%)	Reaction conditions
	GLYA	GLYOA	OA	FA	Others		
(α-)MnO_2-P	17.4	30.6	19.5	24.1	8.1	31.6	120°C 6h
(α-)MnO_2-T	3.8	8.2	10.7	67.5	8.8	78.7	120°C 6h
(α-)MnO_2-D	1.4	3.2	2.8	83.2	8.8	99.2	120°C 6h
(β-)MnO_2-H	22.5	11.8	5.8	27.1	30.2	39.1	120°C 6h
(β-)MnO_2-L	10.5	5.7	2.5	63.5	15.9	79.0	120°C 6h
(γ-)MnO_2-H	15.2	9.1	10.6	26.2	37.1	41.4	120°C 6h
(γ-)MnO_2-L	12.5	0.0	5.3	56.5	23.9	80.7	120°C 6h
Only Glycerol	Glycerol conversion: 23.8%			-			120°C 0.5h
Methanol/Glycerol=0.5	Glycerol conversion: 8.9%			Methanol conversion: 34.4%			120°C 0.5h
Isopropanol/Glycerol=0.5	Glycerol conversion: 14.7%			Isopropanol conversion: 54.8%			120°C 0.5h
Tert butanol/Glycerol=0.5	Glycerol conversion: 11.5%			Tert butanol conversion: 20.0%			120°C 0.5h
Na_2CO_3/Glycerol=0.5	Glycerol conversion: 13.0%			-			120°C 0.5h
Na_2HCO_3/Glycerol=0.5	Glycerol conversion: 14.5%			-			120°C 0.5h
$Ce_2(CO_3)_3$/Glycerol=0.5	Glycerol conversion: 5.7%			-			120°C 0.5h
MnO_2-D(base free)-1	0.5	4.1	2.0	68.9	23.1	29.1	120°C 6h
MnO_2-D(base free)-2	0.9	2.3	3.8	61.1	29.5	30.5	120°C 6h

Note: other reaction conditions: 0.1 g catalyst, 25 mL aqueous solution of glycerol (0.1 M), 0.5 g NaOH, 1 MPa O_2.

The main function of introducing NaOH is to polarize the metal surface (M–OH*) in the initial reaction stage. Supplementary Table 6-2 shows the evaluation experiments of MnO_2-D under base-free conditions. Although FA is still the main product, MnO_2-D needs to react for 6 hours to achieve a conversion rate of ~30% (equivalent to the result under alkaline conditions for 1 hour). This is mainly because the M-OH* generated by the dissociation of O_2 and H_2O needs to reach surface equilibrium to promote the activation of intermediate (for example, aldehydes are attacked by M–OH* to form carboxylic acid like intermediates) and consume H removed from C–H bond and O–H bond to re-activate the metal oxide sites, which has been confirmed by many previous reports. In addition, the introduction of base could also maintain the presence of formic acid in the form of sodium formate, avoiding the decomposition of the target product above 100 ^{o}C.

Supplementary Table 7. Oxidation of glycerol over MnO_2-D catalysts and reported catalysts

	Catalyst	Selectivity (%)				Conversion (%)	TOF (h^{-1})	Reaction conditions	Reference (Year)
		GLYA	GLYOA	TA	FA				
**MnO_2-D+O_2**	MnO_2-D	1.4	3.2	0.0	83.2	99.2	113.5	120ºC, 1Mpa, 8 h	This work
**Noble metals + O_2**	Pt_1/HAP	90.3	1.8	7.8	0.0	91.2	351.2	50ºC, 1MPa, 16 h	[1] (2022)
	AuCu/ZnO	3.6	1.1	-	-	3.1	75.4	60ºC, 1MPa, 8 h	[2] (2022)
	Pt_1+Pt_n/Cu-CuZrO_x	80.0	1.1	1.4	0.0	90.1	224.0	60ºC, 1bar, 8 h	[3] (2022)
	Pt/MgO/SAB-15 (0.1)	65.5	5.4	2.0	0.9	60.2	1671.2	60ºC, 1MPa, 8 h	[4] (2019)
	Pt/SAB-15	49.9	5.0	2.0	0.1	25.5	121.0	60ºC, 1MPa, 8 h	[4] (2019)
**Non noble metals + O_2**	$Cu_1Mg_4O_x$	8.9	60.3	0.1	22.0	89.2	0.2	200ºC, 1MPa, 4 h	[5] (2022)
	Mn_2O_3-T	9.8	41.7	-	-	68.6	0.4	140ºC, 1MPa, 12h	[6] (2021)
	$Ni_1Co_1O_x$	66.5	19.5	-	6.9	78.5	5.4	80ºC, 1MPa, 24 h	[7] (2020)
	$Co_{0.15}$/Mg_3Al-c	29.2	-	46.9	13.4	1.1	0.7	70ºC, 1MPa, 24 h	[8] (2016)
	$Co_{0.15}$/Mg_3Al-s	2.1	-	63.5	2.4	100	2.4	70ºC, 1MPa, 24 h	[8] (2016)
	V_3/ZrP-m	-	-	-	50.9	89.0	0.7	170ºC, 3MPa, 4 h	[9] (2019)
**Heterogeneous catalyst +H_2O_2**	CuN_4/NCSWCs	0.0	6.4	0.0	61.3	96.3	1.1	80ºC, 8 h	[10] (2022)
	Au-PTA/MSN	-	-	-	79.2	33.6	7.1	80ºC, 24 h	[11] (2021)
	MCM-41	-	-	-	36.1	45.0	248.0	150ºC, 8 h	[12] (2019)
	BiOCl/CuNiAl	3.3	5.6	-	11.9	75.4	12.8	60ºC, 6 h	[13] (2019)

[1] H. Yan et al. Angew. Chem. 2022, e202116059.

(Reaction conditions: glycerol[glycerol/Pt molar ratio=250, 25 mL glycerol solution(0.05M), 0.5 g NaOH, 50°C, 1 MPa O_2, 16 h)

[2] Zhao M et al. AICHE, 2022, 16, 34.

(Reaction conditions: 20 mL aqueous solution of GLY (0.105 M), 0.1 g solid catalysts, 60°C, 5 h)

[3] An Z et al. Nature Comm, 2022, 13, 5467.

(Reaction conditions: 15 mL aqueous solution of GLY (0.10 M), Glycerol/metal=300, 60°C, 8 h)

[4] H. Yan et al. J Catal. 370 (2019), 434-449.

(Reaction conditions: 0.2 catalyst, 25 mL aqueous solution of glycerol (0.22 M), glycerol/Pt molar ration 530, 60 ºC, 1 MPa O_2, 8 h.)

[5] Xu S, Xiao Y, Zhang W, et al. Chem. Eng. J, 428 (2022) 132555.

(Reaction conditions: 0.2 g glycerol; 473 K; 0.24 g MgO or 0.16 g MgO; 1 MPa O_2; 400 rpm; 50 mL H_2O)

[7] Yan H, Shen Q, Sun Y, et al. ACS Catal. 2021, 11, 6371−6383.

(Reaction conditions: 0.05 g catalyst, 25 mL aqueous solution of glycerol (0.105 M), 140℃, 1MPa O_2, 12h)

[8] X. Jin, M. Zhao, C. Zeng, et al. ACS Catal. 6 (2016) 4576-4583.

(Reaction conditions: 0.5 g glycerol, 1.5 g NaOH, 25 mL, 0.2 g solid catalysts. “Others”: lactic, glycolic and formic acids, 24 h)

[9] D. Li, H. Gong, et al. Mol. Catal. 474 (2019) 110404.

(Reaction conditions: 5 mL glycerol aqueous solution (10 wt.%), 25 mg catalyst, 170 °C, 4 h, 3 MPa O_2)

[10] D. Li, H. Gong, et al. ACS Sustainable Chem. Eng. 10 (2022) 17177-17186.
(Reaction conditions: 10 mL glycerol aqueous solution (0.1 M), 20 mg catalyst, 80 °C, 8 h, $NaHCO_3$/GLY = 4, H_2O_2/GLY=4)
[11] Lin Y, Yang J, Mou C. ACS Sustainable Chem. Eng. 2021, 9, 3571−3579
(Reaction conditions: 5 mL of 0.6 mol/L glycerol, glycerol/H_2O_2 molar ratio of 1:5, catalyst amount = 10 mg, 80 °C, 24 h)
[12] Lin Y, Yang J, Mou C. ACS Sustainable Chem. Eng. 2021, 9, 3571−3579
(Reaction conditions: 3mL 50%H_2O_2, space velocity of 59.7 h^{-1}, 150 °C, 8 h)
[13] X. Wang, G. Wu, X. Zhang, D. Wang, J. Lan, J. Li, Catal. Lett. 149 (2019) 1046-1056.
(Reaction conditions: 25 mL glycerol aqueous solution (0.2 mol/L), 0.2 g catalyst, 60 °C, 3ml 20%H_2O_2, 6 h)

In order to compare the catalytic performance of MnO_2-D for glycerol oxidation with other reported catalysts, we've categorized the most recently reported catalysts related to this reaction system into three groups: (1) noble metals + O_2 (with other keto acids being the main product), (2) non-noble metals + O_2, and (3) heterogeneous catalyst + H_2O_2.

From the first group (1), we observe that MnO_2-D's catalytic activity in glycerol oxidation using O_2 approaches the levels achieved by precious metals. From the second group (2), it's evident that MnO_2-D delivers the highest catalytic activity and FA selectivity among the non-precious metals using O_2. The third group (3) showcases MnO_2-D's capability to activate O_2, demonstrating performance superior to heterogeneous catalysts utilizing H_2O_2 as an oxidant.

Kinetics determination

I. Elimination of the diffusion limitations

The MnO_2-D catalyst was selected as a sample to investigate the internal and external mass transfer, and the oxidation of glycerol (GLY) to formic acid (FA) was used as a model reaction. It is ensured that all the catalysts were evaluated with negligible mass transfer limitations.

(1) Effect of external diffusion on GLY oxidation

(Reaction conditions: 120 °C, 1MPa O_2, 0.1M Glycerol, 0.1 g Cat.)

(a) At 700 RPM, (5 min) $R_{oxidation} = 0.120\ kmol/m^3 \cdot h$;

(b) At 1000 RPM, (5 min) $R_{oxidation} = 0.125\ kmol/m^3 \cdot h$;

(c) At 1200 RPM, (5min) $R_{oxidation} = 0.118\ kmol/m^3 \cdot h$;

(2) Interphase Mass Transfer Limitation for Oxygen

(a) Gas-Liquid Mass Transfer Limitation: (Three phase catalytic reactors, Ramachandran & Chaudhari, 1983; J. Chem Eng Data. 1984, 29, 286-287; Chem. Eng. Process, 2004, 43, 823-830; J. Catal. 2016, 337, 272-283; J. Catal. 2008, 257, 1-4)

$$\frac{R_{oxidation} d_{bubble}}{6 \cdot \varepsilon \cdot k_{g\text{-}l} \cdot C_{o_{2,\,b}}} = \frac{0.121\ (kmol/m^3 \cdot h) \cdot 0.000002 (m)}{6 \cdot 0.09 \cdot 1.44 (m/h) \cdot 0.01 (kmol/m^3)} = 3.19 \times 10^{-5} < 0.1$$

(b) Liquid-Solid Mass Transfer Limitation: (J. Catal. 2016, 337, 272-283; Three phase catalytic reactors, Ramachandran & Chaudhari, 1983; HAP, assume 100 nm, see Fig. S8)

$$\frac{R_{oxidation} \rho_p \cdot d_p}{6 \cdot \omega_{cat} \cdot k_{l\text{-}s} \cdot C^*_{o_{2,\,b}}} = \frac{0.121 (kmol/m^3 \cdot h) \cdot 2000 (kg/m^3) \cdot 40 \cdot 10^{-7} (m)}{6 \cdot 8 (kg/m^3) \cdot 72.1 (m/h) \cdot 0.01 (kmol/m^3)} = 2.86 \times 10^{-6} < 0.1$$

(c) Internal Diffusion: (J. Catal. 2016, 337, 272-283; Three phase catalytic reactors, Ramachandran & Chaudhari, 1983; Perry's Handbook: Table 5-16)

$$\frac{R_{oxidation} \rho_p \cdot d_p^2}{4 \cdot \omega_{cat} \cdot D_e \cdot C^*_{o_{2,}}} = \frac{0.121 (kmol/m^3 \cdot h) \cdot 2000 (kg/m^3) \cdot (40 \cdot 10^{-7} m)^2}{4 \cdot 8 (kg/m^3) \cdot 0.094 (m^2/h) \cdot 0.01 (kmol/m^3)} = 1.35 \times 10^{-8} < 1$$

(3) Interphase Mass Transfer Limitation for Glycerol

(a) Liquid-Solid Transfer Limitation: (J. Catal. 2016, 337, 272-283; AIChE J. 1980, 26, 177-201; Three phase catalytic reactors, Ramachandran & Chaudhari, 1983)

$$\frac{R_{oxidation} \rho_p \cdot d_p}{6 \cdot \omega_{cat} \cdot k_{l\text{-}s} \cdot C_{gly}} = \frac{0.121 (kmol/m^3 \cdot h) \cdot 2000 (kg/m^3) \cdot 40 \cdot 10^{-7} (m)}{6 \cdot 8 (kg/m^3) \cdot 0.6 (m/h) \cdot 0.1 (kmol/m^3)} = 3.4 \times 10^{-5} < 0.1$$

(b) Intraparticle Transfer Limitation: (J. Catal. 2016, 337, 272-283; AIChE J. 1980, 26, 177-201; Three phase catalytic reactors, Ramachandran & Chaudhari, 1983)

$$\frac{d_p}{6} \left[\frac{(m+1) \cdot R_{oxidation} \rho_p}{2 \cdot \omega_{cat} \cdot D_e \cdot C_{gly}} \right]^{0.5} = \frac{10^{-7} (m)}{6} \left[\frac{0.121 (kmol/m^3 \cdot h) \cdot 2000 (kg/m^3)}{8 \cdot (kg/m^3) \cdot 0.036 (m^2/h) \cdot 0.1 (kmol/m^3)} \right]^{0.5} = 1.5 \times 10^{-6} < 0.2$$

3. Supplementary Figures

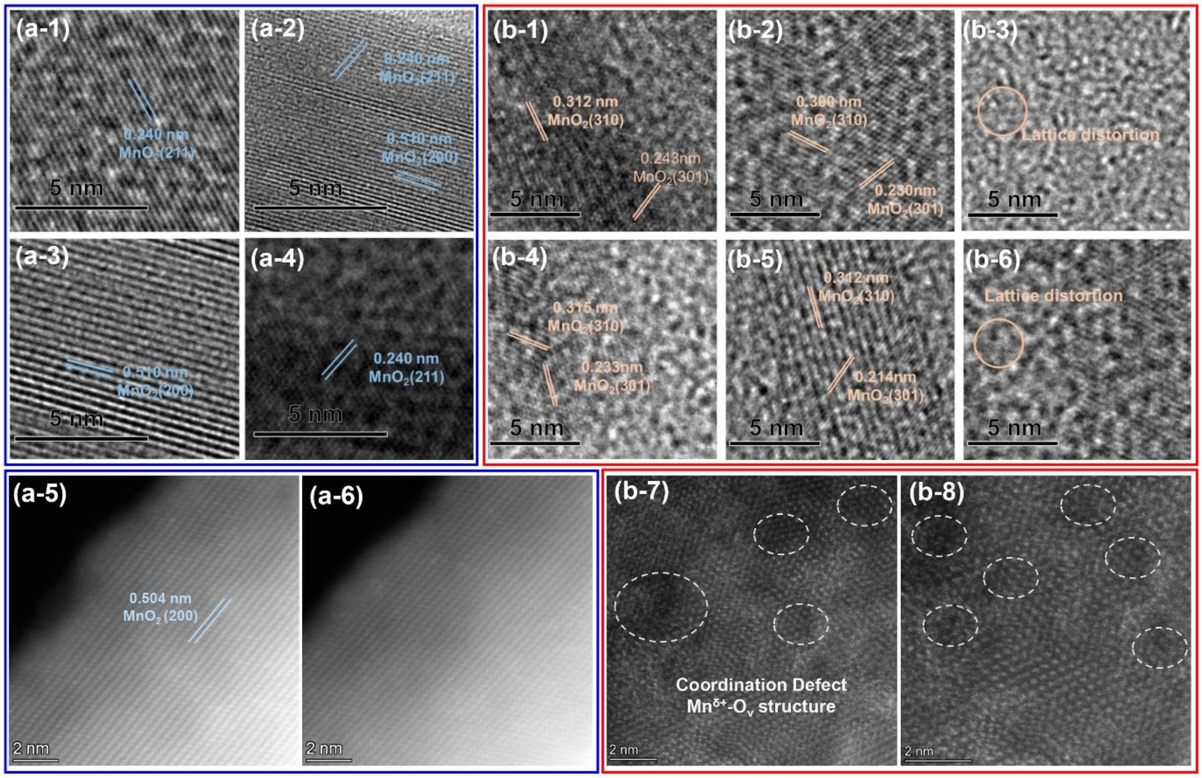

Supplementary Fig. 1 Supplemental HRTEM images for the (a) MnO_2-P and (b) MnO_2-D catalysts.

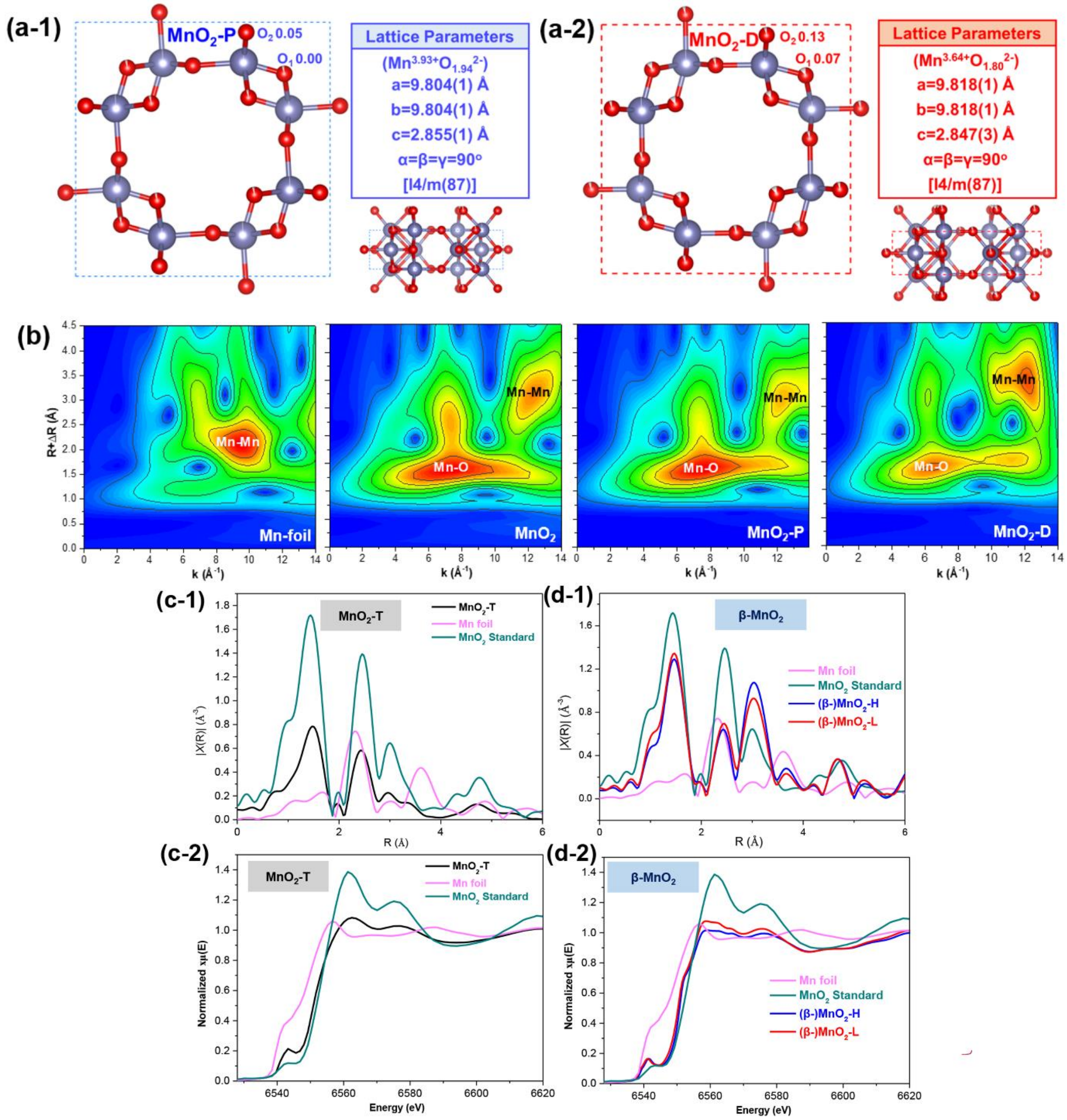

Supplementary Fig. 2-1 (a) Visualisation of the associated crystal structure. (b) Wavelet transform map of different coordination atoms. Fourier transform of Mn K-edge extended EXAFS oscillations and the first-order derivatives of Mn K-edge XANES of (c) MnO_2-T and (d) β-MnO_2.

Unit cell of samples MnO_2-P (I4/m (84), a=b=9.804(1) Å, c=2.855(1) Å) and MnO_2-D (I4/m (84), a=b=9.818(1) Å, c=2.847(1) Å) obtained from powder diffraction data based on the Rietveld profile refinement and Stephens peak shape function using the Topas software (Supplementary Table 1-2) with the R_{wp} are 11.62% and 9.82%, respectively.

EXAFS in Supplementary Fig. 2-1 and Supplementary Table 3-1 proves that the average coordination number of MnO_2-T (α phase) catalyst is 4.2, indicating that there is mainly Mn with low coordination number, similar as in defective α-MnO_2 (MnO_2-D). On this basis, the coordination number of MnO_2-P, MnO_2-T and MnO_2-D is 5.2, 4.2 and 3.0, respectively. Further evaluation of glycerol oxidation on this catalyst under the same reaction conditions revealed that the conversion of MnO_2-P, MnO_2-T and MnO_2-D are 31.6%, 78.7% and 99.2%, respectively. Moreover, the FA selectivity also increases with the decrease of coordination number. Obviously, this strategy of reducing the coordination number of α-MnO_2 is effective for improving the catalytic performance of glycerol oxidation.

Then, to further prove the universality of this strategy, we prepared two other common crystalline phases of β-MnO_2 and γ-MnO_2 with high and low coordination number for glycerol oxidation. By reducing the hydrothermal temperature (or changing the synthetic method) during the synthesis process, the crystallinity of the two catalysts can be reduced to decrease the coordination number of Mn. For the β-MnO_2, XRD in Supplementary Fig. 2-2 confirms that both high-coordination (β-)MnO_2-H and low-coordination (β-)MnO_2-L are β crystal phase. EXAFS in Supplementary Table 3-2 and Supplementary Fig. 2-1 proves that the coordination number of (β-)MnO_2-H and (β-)MnO_2-L are 5.4 and 3.4 respectively. The acidity and alkalinity of (β-)MnO_2-L with low coordination number are also enhanced, indicating the presence of FLPs (Supplementary Fig. 2-2). Moreover, (β-)MnO_2-L exhibits higher glycerol conversion and FA selectivity than (β-)MnO_2-H (Supplementary Table 6-2). Similarly, for the γ-MnO_2, (γ-)MnO_2-H has a higher crystallinity, while (γ-)MnO_2-L has a lower crystallinity. (γ-)MnO_2-L also displays higher glycerol conversion and FA selectivity than (γ-)MnO_2-H.

The above results fully show that the strategy of reducing coordination number to promote the oxidation of polyol to formic acid is universal in MnO2 materials with different crystal phases.

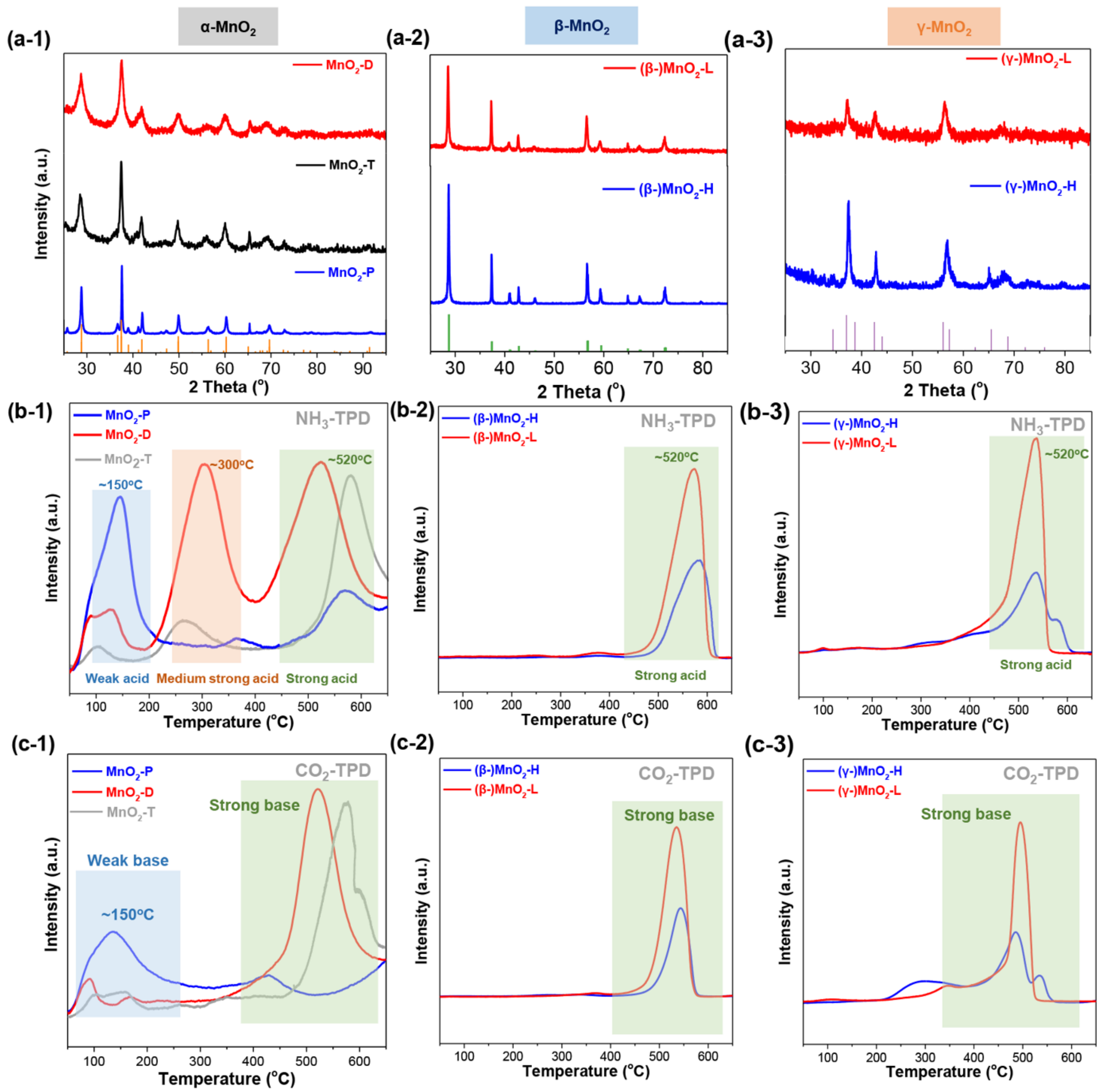

Supplementary Fig. 2-2. (a) XRD patterns, (b) NH_3-TPD and (c) CO_2-TPD of α-MnO_2, β-MnO_2 and γ-MnO_2 with high or low coordination number.

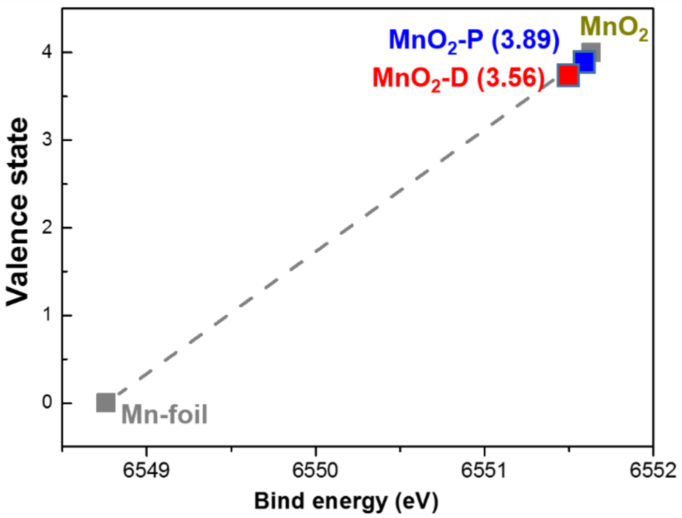

Supplementary Fig. 3 Mn electronic structure analysis from XANES spectra.

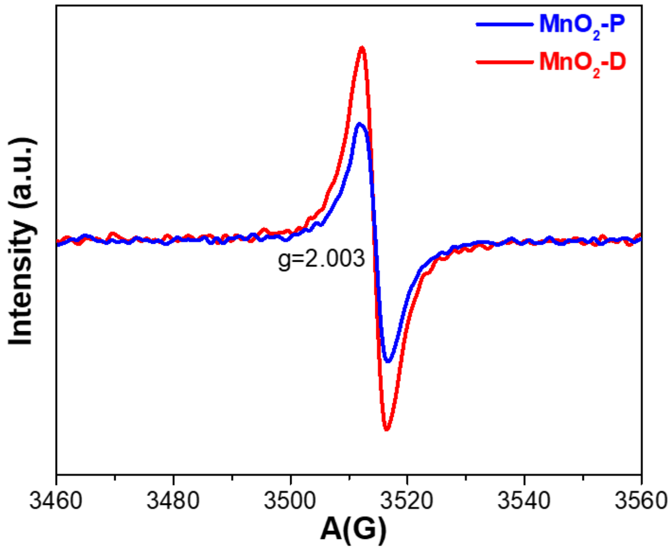

Supplementary Fig. 4 EPR spectra of the MnO_2-P and MnO_2-D catalysts.

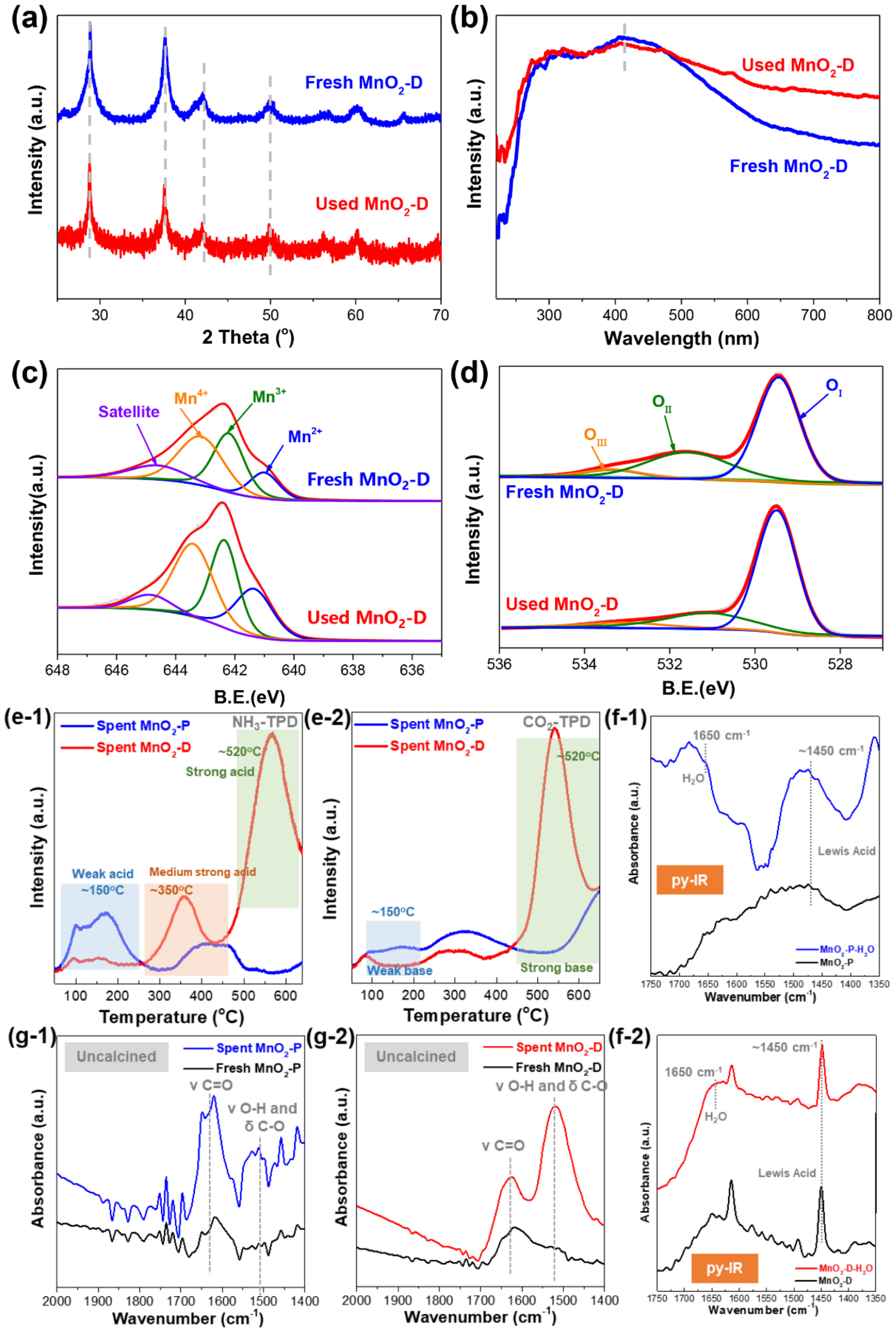

Supplementary Fig. 5 (a)XRD, (b) UV-vis, (c) Mn 2*p* and (d) O 1*s* XPS spectra of the fresh MnO_2-D and used MnO_2-D catalysts. (e) NH_3-TPD and CO_2-TPD of spent MnO_2-P and MnO_2-D. (f) Pyridine (py-)IR of MnO_2-P and MnO_2-D with and without water. (g) IR spectra of the spent MnO_2-P, spent MnO_2-D, fresh MnO_2-P and spent MnO_2-D.

Meanwhile, we further supplemented the NH_3-TPD and CO_2-TPD of spent MnO_2-D. Supplementary Fig.5e shows that the acid and basic properties of the spent MnO_2-D (re-calcined) are not significantly different from those

of the fresh MnO_2-D. There is only a slight decrease in medium strong acid in spent MnO_2-D due to the slight damage of partial structure under long-term reaction conditions. Moreover, MnO_2-D still exhibits stronger and more acid-base sites than MnO_2-P. On these foundations, it can be concluded that the acid and base sites formed in low-coordination MnO_2-D exhibit good catalytic stability in the aqueous oxidation reaction.

Supplementary Fig. 5f shows that both MnO_2-P and MnO_2-D exhibit the peaks at 1450 cm^{-1} belonging to Lewis acid, and MnO_2-D displays stronger L acid peak. Meanwhile, in order to investigate the water tolerant of L acid, MnO_2-P and MnO_2-D with pre-added water were tested under non dehydrated conditions. It is found that the intensity of the L acid peak of MnO_2-D+H_2O at 1450 cm^{-1} is almost unaffected, and even the L acid of MnO_2-P+H_2O is slightly enhanced. Thus, the Lewis acid sites in the MnO_2-P and MnO_2-D are water tolerant.

The main reason for the drop of 10% yield is that some carboxylic acid products gradually cover the surface of the catalyst, resulting in the deactivation of certain sites. To prove this point, we conducted IR characterization of MnO_2-D before and after the reaction. Supplementary Fig. 5g shows that both spent MnO_2-P and MnO_2-D exhibit stronger peaks of stretching vibration of C=O and O–H bonds and deformation vibration of C–O bond compared to fresh catalysts, indicating that the ketone acids (and their condensation products) could partially cover the surface of the catalyst after the reaction. This resulted in a partial decrease in the FA yield of MnO_2-D. After the re-calcination of spent catalyst, the carboxylic acid species covered on the MnO_2-D surface could be removed, thus restoring the catalytic performance.

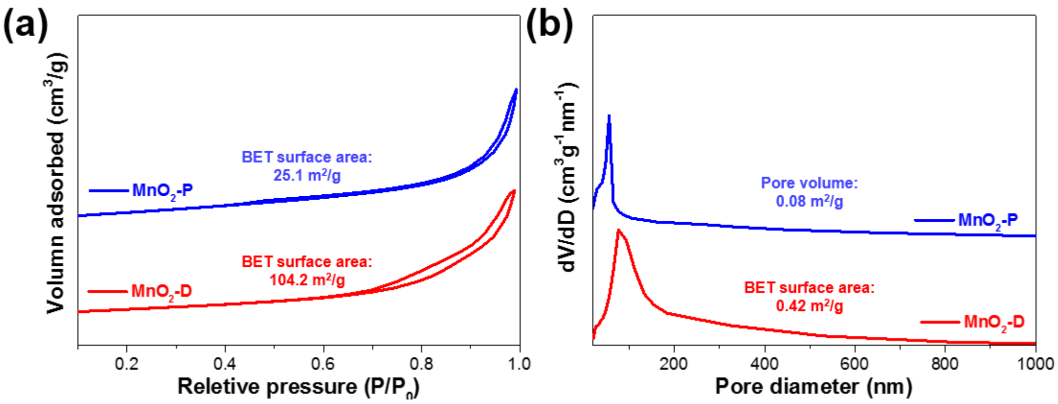

Supplementary Fig. 6 (a) Nitrogen adsorption-desorption isotherms and (b) pore size distributions of the MnO_2-P and MnO_2-D catalysts.

To eliminate the influence of specific surface area on catalytic activity, the quality of the MnO_2-P catalyst 4 times that of the MnO_2-D catalyst was added in the process of catalyst evaluation [Reaction conditions: 0.4 g MnO_2-P catalyst or 0.1 g MnO_2-D catalyst, 25 mL aqueous solution of glycerol (0.1 M), 120 °C, 1 MPa O_2, 6 h]. The conversion of the MnO_2-P and MnO_2-D catalysts are 85.5 and 99.2% respectively, and the selectivity of formic acid on the MnO_2-P catalyst is only 10.5%, far lower than that of the MnO_2-D catalyst (83.2%).

Additionally, we determined the initial reaction rates of MnO_2-D and MnO_2-P catalysts at short reaction times (5 min for both) and low conversion rates, yielding 0.301 and 0.091 mmol/h/m^2 (normalized by specific surface area), respectively. It is clear that the activity of the MnO_2-P catalyst is markedly lower than that of the MnO_2-D catalyst. This suggests that the specific surface area is not the primary factor influencing catalytic performance. Instead, the Mn-O active sites with varied coordination structures, formed by the MnO_2-P and MnO_2-D catalysts, appear to be the critical factor affecting the oxidation of polyols to formic acid.

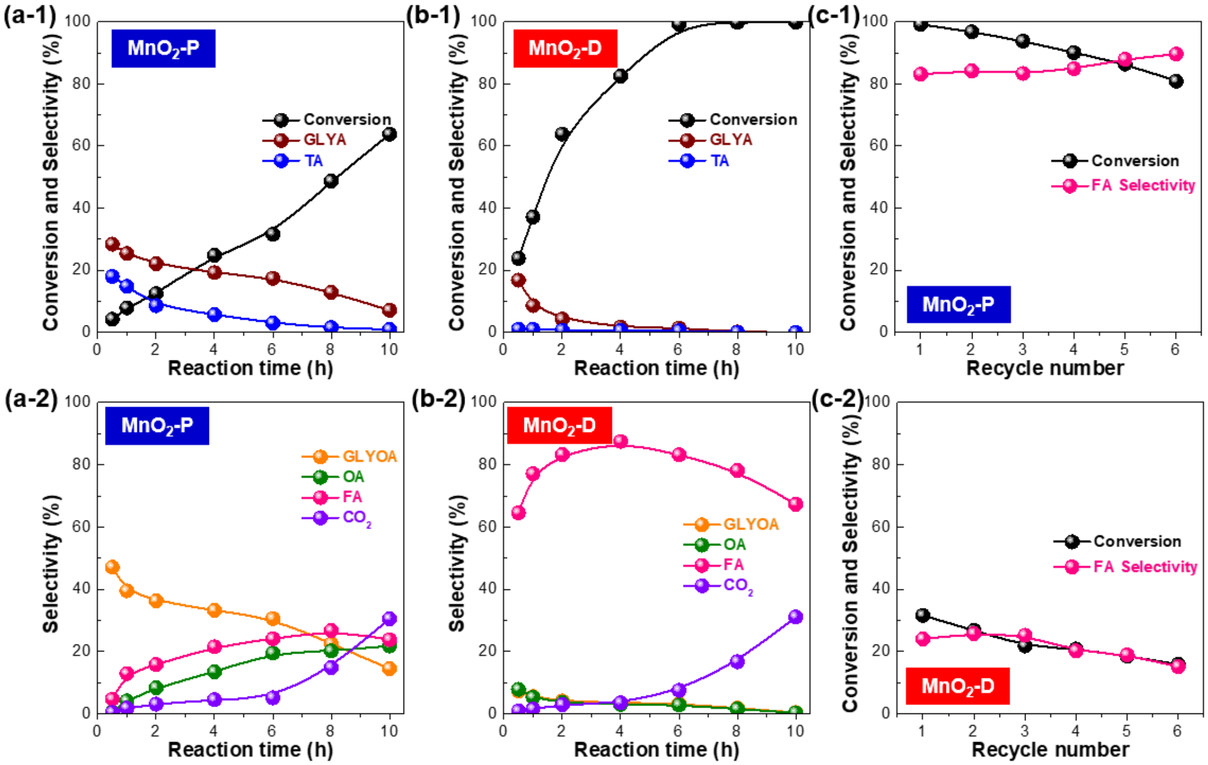

Supplementary Fig. 7 Catalytic performance of glycerol oxidation on (a) MnO_2-P and (b) MnO_2-D catalysts as a function of reaction time (experiment conditions: 25 mL aqueous phase solution (0.1 M), 0.5 g NaOH, 0.1 g catalyst, 1 MPa O_2, 120°C; Glyceric acid, propanedioic acid, glycolic acid, oxalic acid and glycerol are abbreviated as GLYA, TA, GLYOA, OA and FA respectively)**. (c) Catalytic stability of the MnO_2-P and MnO_2-D under multiple cycle test conditions.**

Fig. S7 shows that MnO_2-P could achieve 63.8% conversion after a long reaction time of 10 h, while MnO_2-D could achieve nearly 100% conversion in just 6 hours. Obviously, MnO_2-D has higher catalytic activity. Meanwhile, there is a significant difference in product distribution between the two catalysts. For MnO_2-D, the main product at the initial stage is formic acid (FA), followed by glyceric acid (GLYA). With the extension of reaction time, the GLYA selectivity decreases, and the FA selectivity first increases and then decreases. The CO_2 selectivity increases with the extension of reaction time. These suggests that the reaction path on MnO_2-D may be the primary hydroxyl oxidation and C-C bond cleavage of glycerol→GLYA→GLYOA→FA→CO_2.

In contrast, for MnO_2-P, the main product is glycolic acid (GLYOA) in a short reaction time, followed by GLYA and tartronic acid (TA). With the extension of reaction time, GLYOA, GLYA and TA selectivity decrease significantly, and oxalic acid (OA) selectivity increases. FA selectivity slightly increases with the extension of reaction time (but always below 30%), and excessive reaction time cannot increase its selectivity. These suggests that the reaction pathway on MnO_2-P is primary/secondary hydroxyl groups and C-C bonds of glycerol→GLYOA+GLYA+TA→OA→FA→CO_2.

Obviously, MnO_2-P tends to activate the hydroxyl groups and C-C bonds indiscriminately, resulting in uneven product distribution and low FA selectivity. These conclusions are consistent with the results of in situ FTIR.

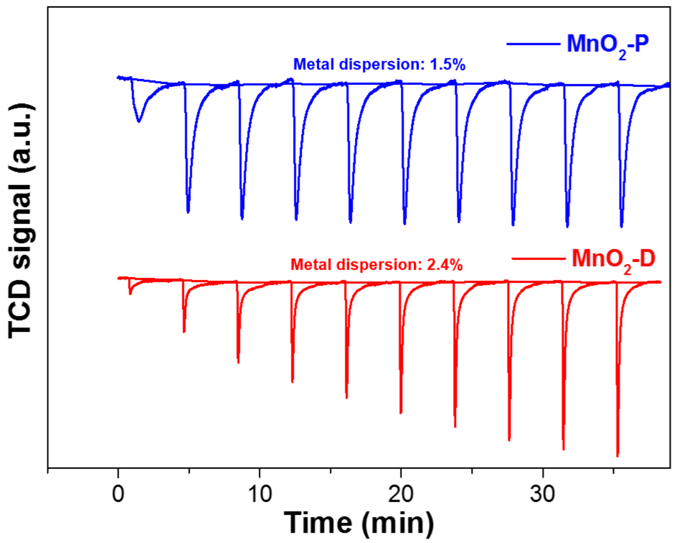

Supplementary Fig. 8 A schematic diagram of ethanol pulse adsorption of the MnO_2-P and MnO_2-D catalysts.

The reason why we chose ethanol instead of glycerol or other polyols as probe molecule was that the boiling point (290 ^{o}C) and desorption temperature of glycerol were very high and it was difficult to produce glycerol gas through steam generator. Selecting ethanol as a model compound can represent the adsorption performance of alcohol oxidation.

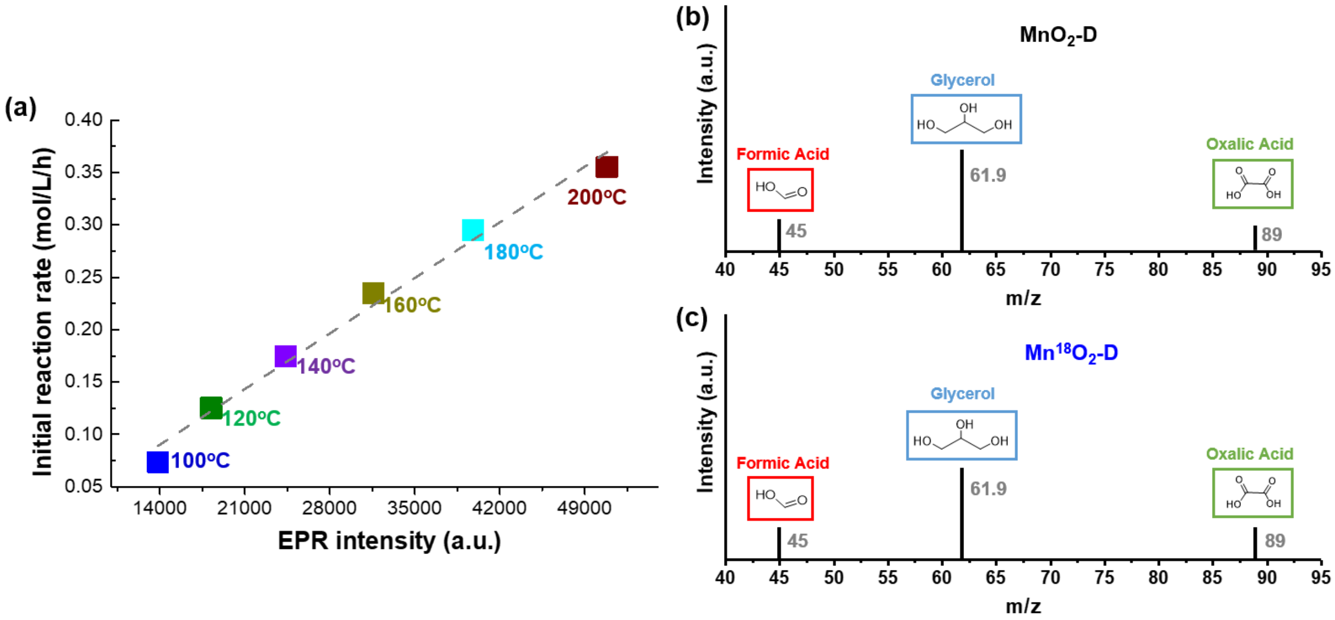

Supplementary Fig. 9 (a) The linear relationship between initial reaction rate (mol/L/h) and EPR intensity. Oxidation mechanism study based on the isotope labelling experiments over (b) MnO_2-D and (c) $Mn^{18}O_2$-D. [Reaction conditions: 25 mL aqueous phase solution (0.1 M), 0.1 g catalyst, 1 MPa O_2, 140°C, 4 h].

Isotope experiments were conducted to investigate whether labelled lattice oxygen in MnO_2-D participates in the oxidation reaction. To prepare the $Mn^{18}O_2$-D, the sample obtained in hydrothermal method are directly calcined under $^{18}O_2$ gas during the preparation process. In addition, the evaluation conditions of the catalyst prohibit the addition of sodium hydroxide to meet the test requirements of LC-MS. The conversion of MnO_2-D and $Mn^{18}O_2$-D is ~30% at 140°C in the base-free medium. The main products are mainly formic acid (~30% selectivity), and the other products are oxalic acid and CO_2. Obviously, Supplementary Fig. 8 shows that there is no significant difference in the peak positions of products on MnO_2-D and $Mn^{18}O_2$-D, indicating that isotope labeled lattice ^{18}O does not participate in the oxidation reaction. In other word, the glycerol oxidation does not involve Mars van Krevelen (MvK) mechanism.

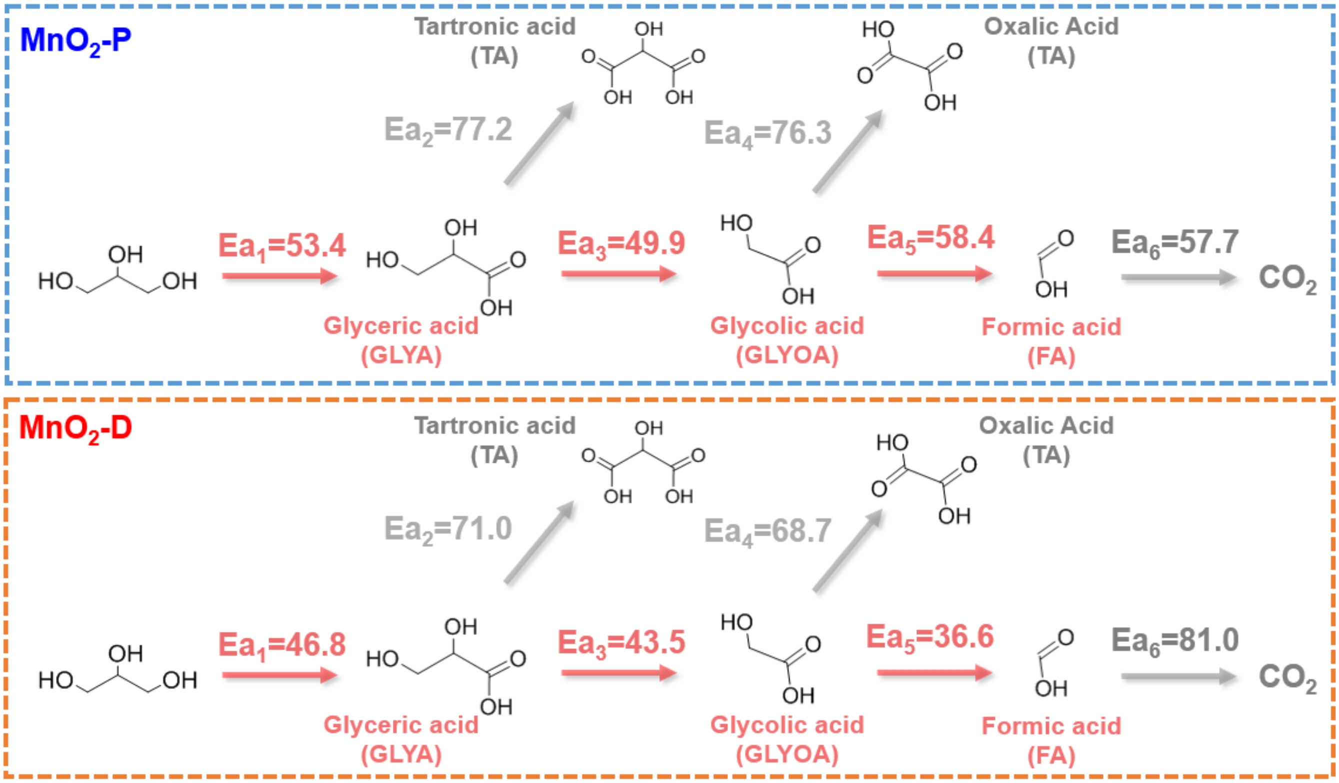

Supplementary Fig. 10 Apparent activation energy calculated from the power function type reaction kinetic equation over the MnO_2-P and MnO_2-D.

II. The results of apparent reaction kinetics

The power function type reaction kinetic equation was used to investigate the oxidation of glycerol over the MnO_2-P and MnO_2-D catalysts. The equation can be expressed as:

$$r_i = -\frac{dC_0}{dt} = A\,exp\left(\frac{-Eai}{RT}\right) C_G^a P_O^b$$

P_O and C_G are the initial concentration of O_2 pressure and glycerol (in mol g_{Pt}^{-1}) respectively; a and b are the corresponding reaction order. T, r, R, A and Eai are the reaction temperature (K), the initial reaction rate of one substrate (in mol g_{Pt}^{-1} h^{-1}), ideal gas constant (8.314×10^{-3} kJ mol^{-1} K^{-1}), the pre-exponential factor and activation energy (kJ mol^{-1}).The concentration of oxygen at the surface equals its bulk concentration (calculated by Henry’s law). [J. Chem. Thermodyn. 2000, 32, 1145; J. Phys. Chem. C, 2010, 114, 1164–1172; J. Phys. Chem. 1996, 100, 5597].The initial reaction rate is almost constant in the range of 1-1.8 MPa O_2, suggesting that the reaction order of oxygen partial pressure could be considered as zero. The reaction order of glycerol (GLY) for the MnO_2-P and MnO_2-D catalysts are 0.49 and 0.41 respectively.

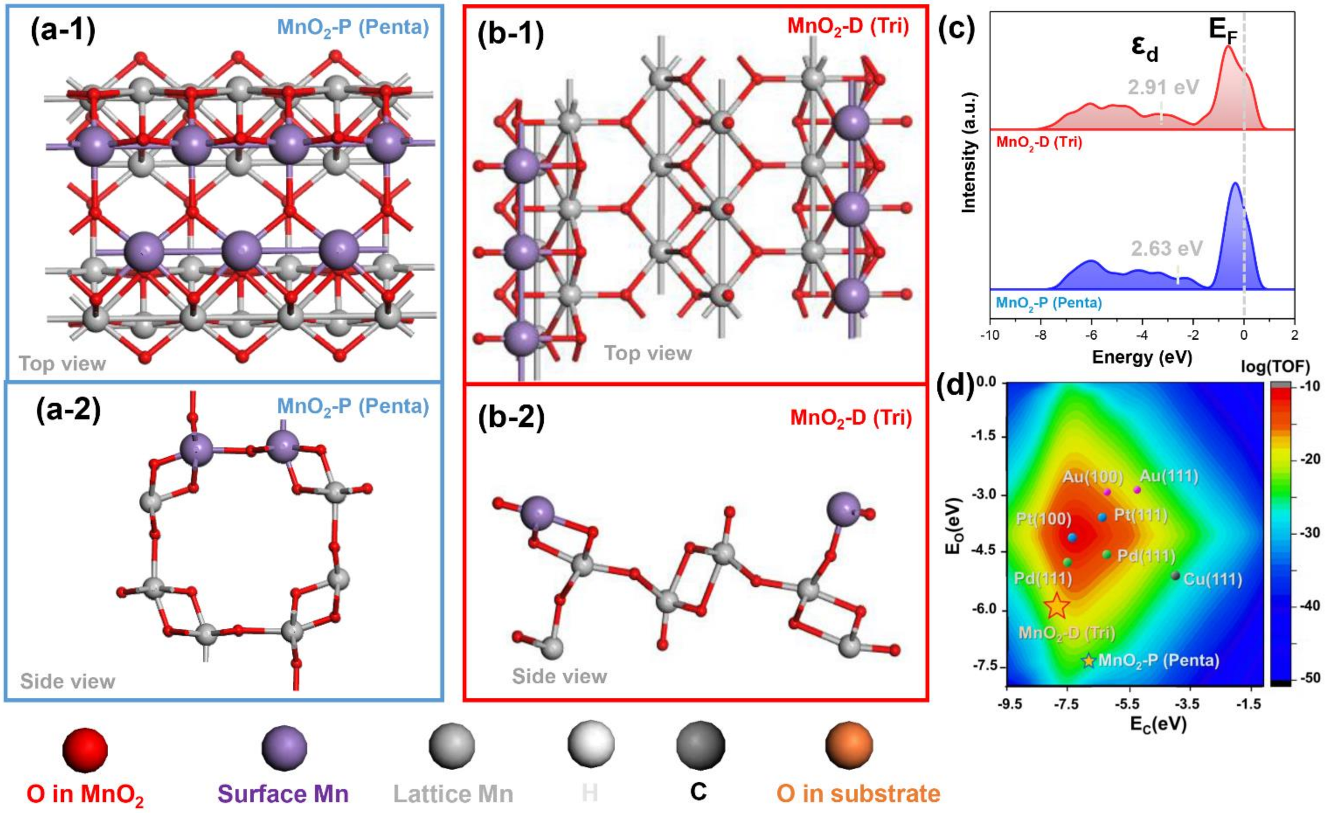

Supplementary Fig. 11 DFT calculation models of (a) the MnO_2-P (Penta) and (b) MnO_2-D (Tri). (c) Partial density of states (PDOS) of MnO_2-P (Penta) and MnO_2-D (Tri). (d) Activity map for glycerol oxidation.

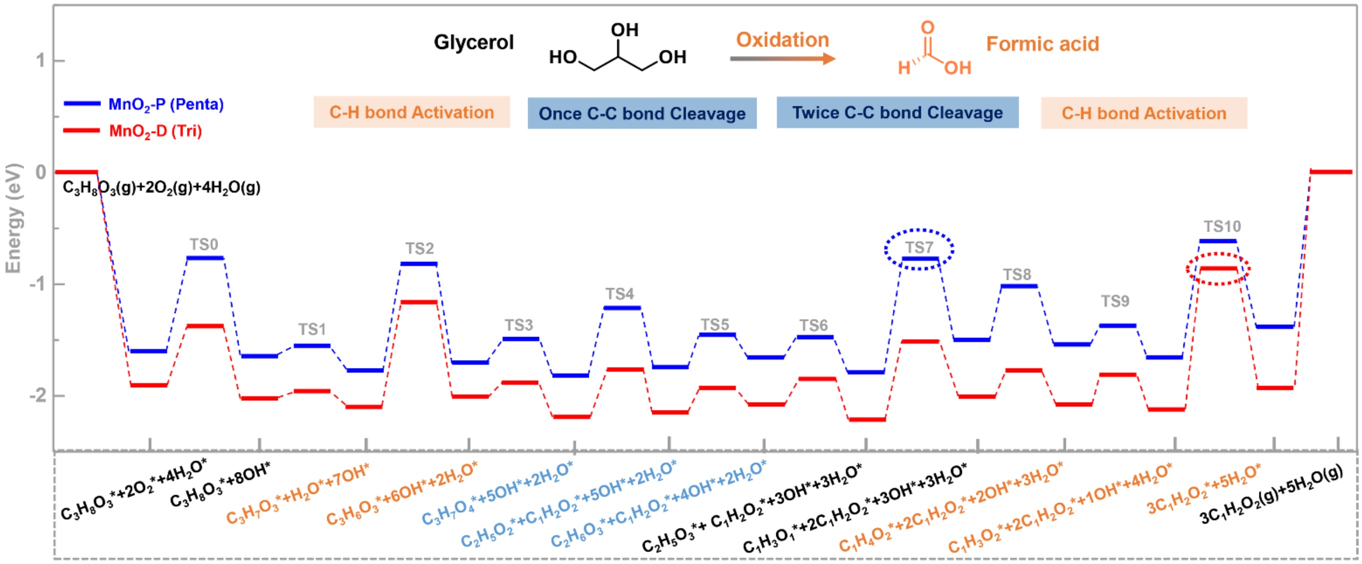

Supplementary Fig. 12-1 Free energy diagrams for the oxidation of glycerol to formic acid on the MnO_2-P (Penta) [blue] and MnO_2-D (Tri) [red]

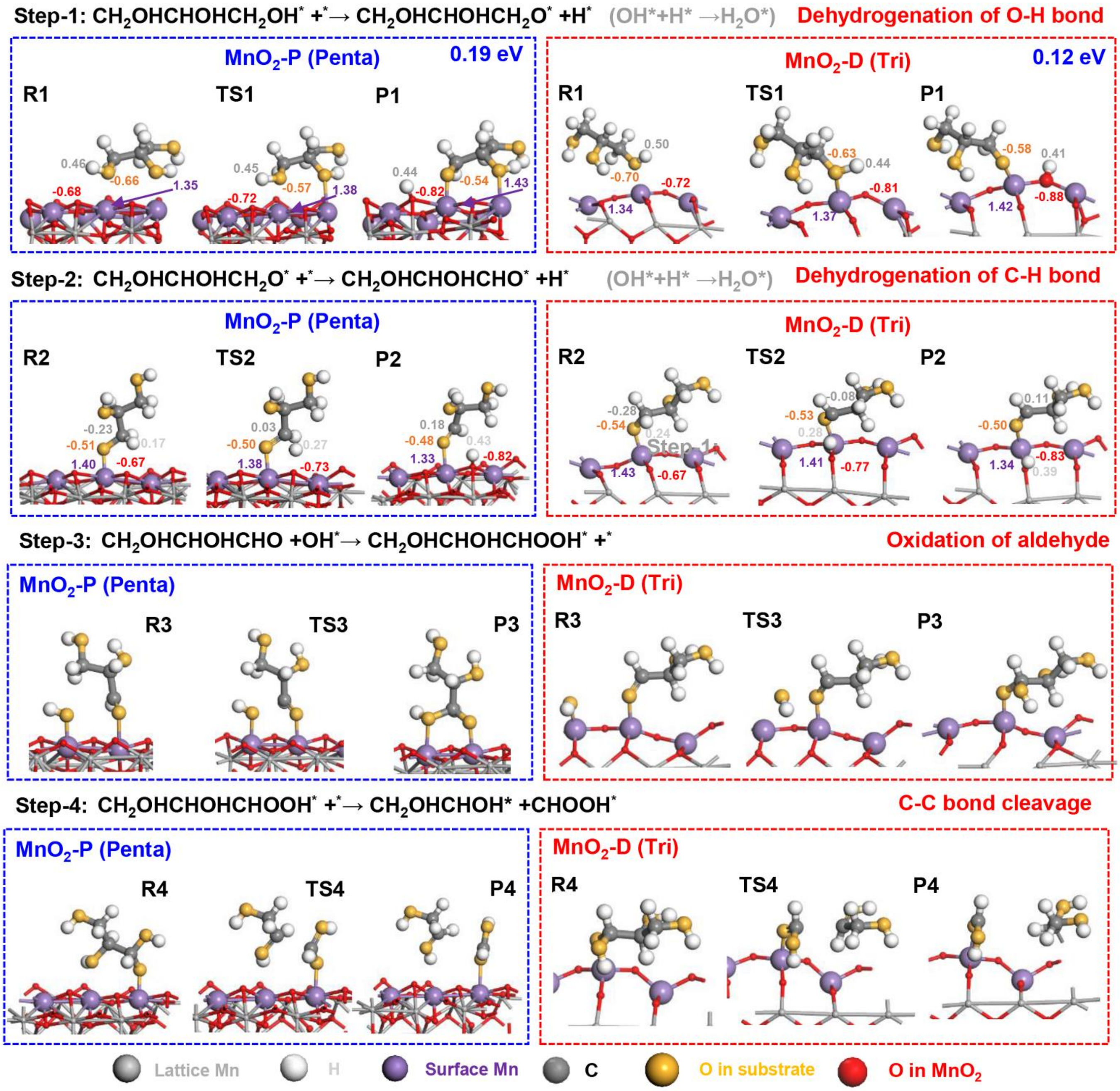

Supplementary Fig. 12-2 Configuration diagrams of reactants, transition states, and products on the MnO_2-P (Penta) [blue] and MnO_2-D (Tri) [red] (Step-1 to Step-4)

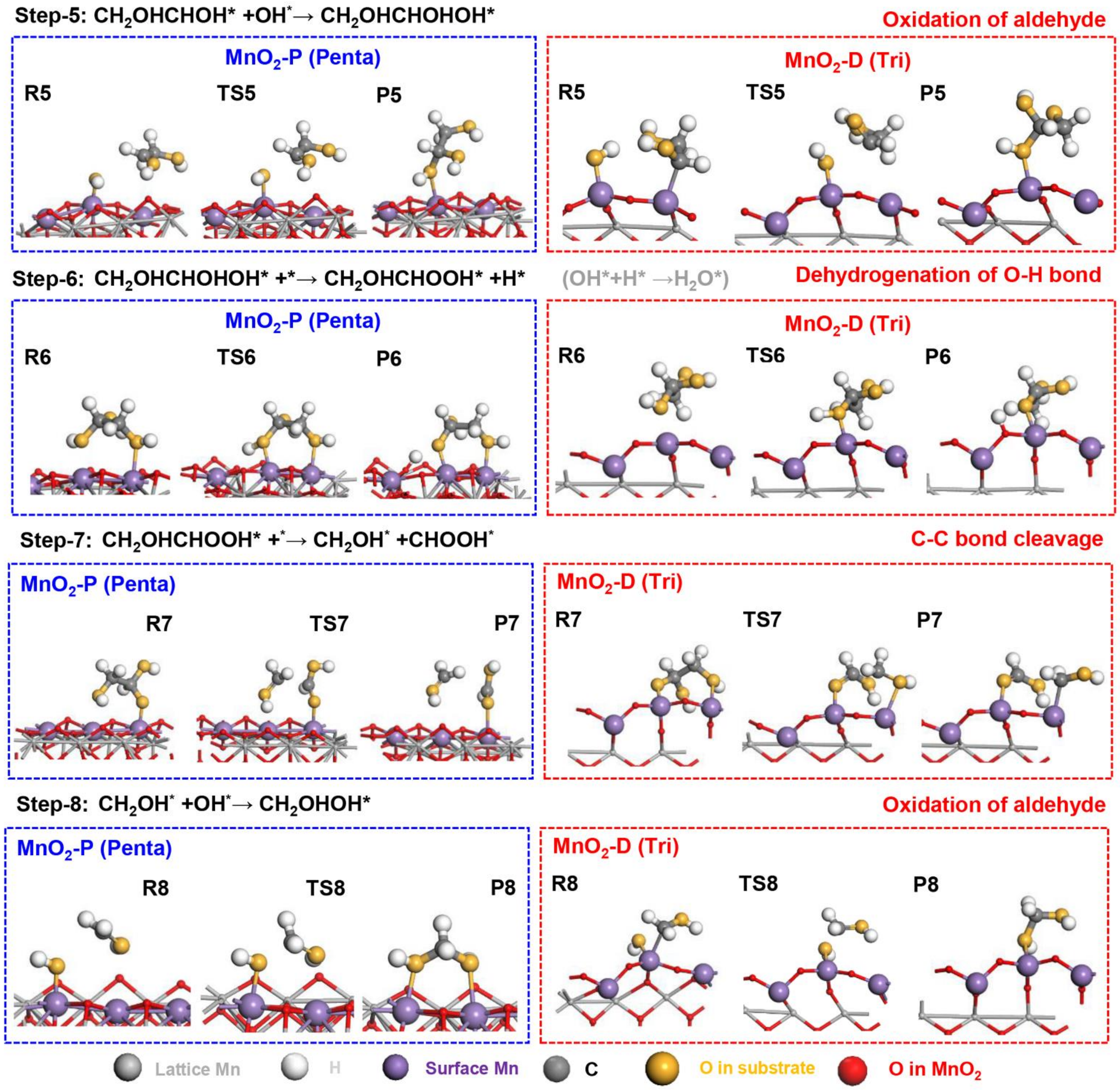

Supplementary Fig. 12-3 Configuration diagrams of reactants, transition states, and products on the MnO_2-P (Penta) [blue] and MnO_2-D (Tri) [red] (Step-5 to Step-8)

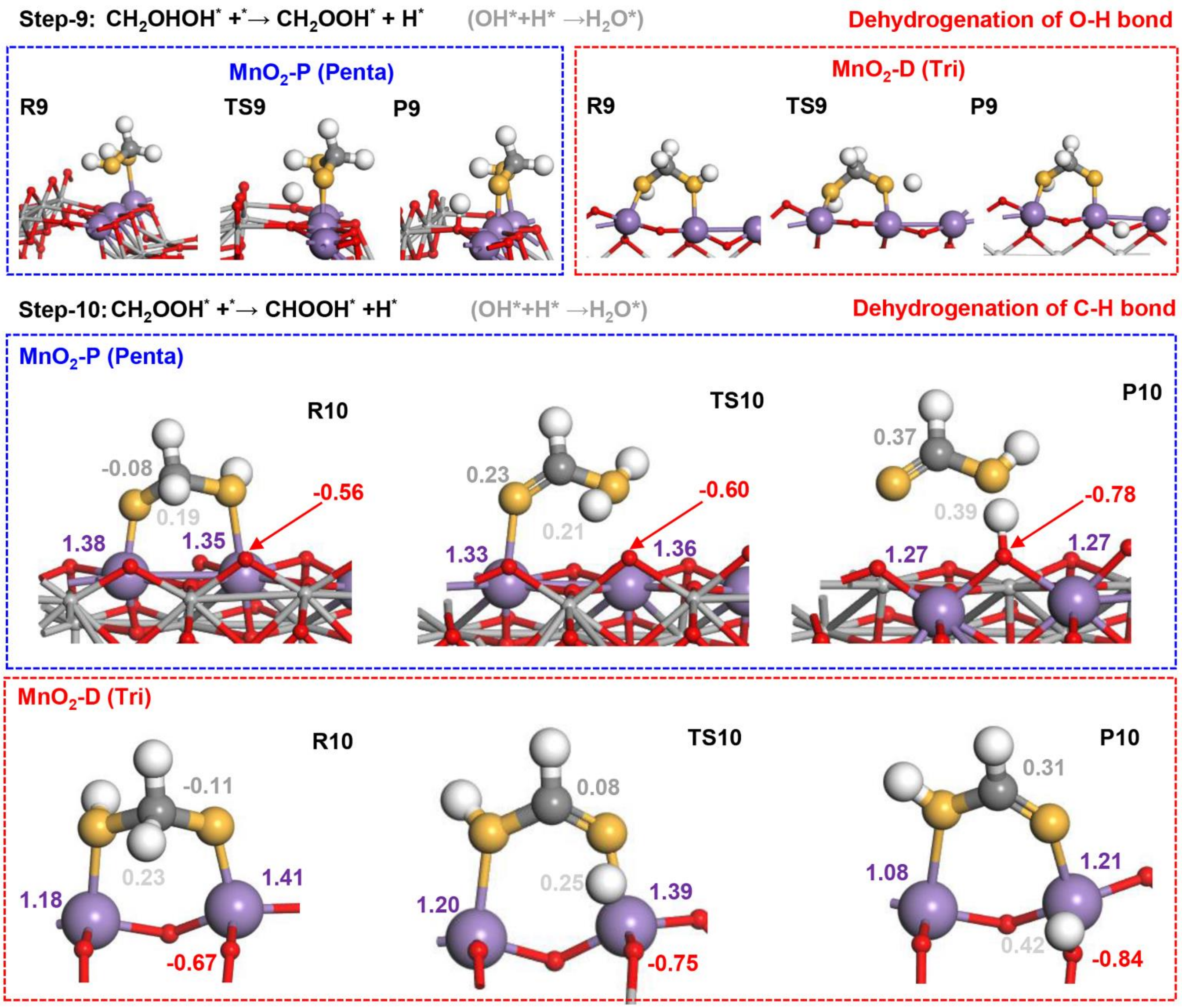

Supplementary Fig. 12-4 Configuration diagrams of reactants, transition states, and products on the MnO_2-P (Penta) [blue] and MnO_2-D (Tri) [red] (Step-9 to Step-10). Mulliken charge (|e|) distribution of the rate-determining step (Step-10) in the MnO_2-P (Penta) and MnO_2-D (Tri).
